# Spending the Summer

**Published:** 1871-04

**Authors:** 


### SPENDING THE SUMMER.

Change is the great health-principle involved, and the more complete that change the better, — change of air, of scene, of occupation, of food, of mode of preparation, of associations; hence the people of the city should go into the country, the deeper into its recesses the better; and persons from the country cannot do better than to spend a month or two or three in the city during warm weather. The city ways would be a constant source of interesting observation and study; would be a means of elevation, of expansion; would give a greater breadth of view of things. Under ordinary favorable circumstances, a man becomes more of a man, and a woman more of a woman, by the contemplation of, and mingling with city life; their views of things become greatly enlarged, while the conduct and the manners are elevated and improved. As a matter of economy to those coming from the country, it is largely in favor of the city, as compared with the seaside, the watering places, and other fashionable resorts; for at all of these the charges vary from two to six dollars a day, which, with inevitable "extras," is increased by fifty per cent., and all this for rooms often so small, that for toilet purposes one must remain in bed or leave the house until the other is dressed; rooms with rickety "cottage furniture," soiled carpets, cloddy beds, bad attendance, and everlasting din, and dust, and dirt, everywhere. In New York City, in any summer, large, elegant rooms, in commodious brown stone houses, with all the appointments of cultivated life, in or near the best streets, can be had for ten dollars a week, easily. A ride to and through the Central Park and return home costs less than half a dollar; for half that amount a person can go to the Park and return from any part of New York, and can spend an hour or two there without any expense, with new faces, and new equipages, and new views, every day and every hour. For a dollar one may take an omnibus to South Ferry, go on a boat down the bay, in sight of the sea, spend a few hours on shore on Staten Island, take a picnic, and return by the time of the setting sun. Between sunrise and dark an excursion can be made to Long Branch, with sea-bathing, a delicious dinner, and a movable, changing, splendid panorama of elegant men, of handsome woman, of beautiful equipages; returning to spend the night in large and airy chambers, with no mosquitoes, no drunken revelry, nor dice, nor cards, nor thimble-riggery, nor noisy halls, nor degrading gallantries.

Then the promenades in Broadway or on Fifth Avenue are a source of never-failing pleasure, amusement, and health; the sidewalks are always dry, and in the hottest days there is a shady side to every street, and a saunter can be made for minutes or for hours, every step something new to meet the eye, something lively to attract the attention. In addition to these are city preaching, lectures, libraries, and reading-rooms. In short, we invite everybody in the country to spend the summer in New York City.

